# Executive Dysfunction and Disability in SPMS: Predictive Value of the Frontal Assessment Battery in the UCLH MS‐STAT2 Cohort

**DOI:** 10.1111/ene.70286

**Published:** 2025-07-09

**Authors:** Charles Wade, Anisha Doshi, Sean Apap Mangion, Tom Williams, Alessia Bianchi, Floriana De Angelis, Sarah Wright, Nevin John, Alberto Calvi, Marie Braisher, James Blackstone, Jennifer Nicholas, Jeremy Chataway, The UCL MS‐STAT2 Investigators

**Affiliations:** ^1^ Queen Square Multiple Sclerosis Centre, Department of Neuroinflammation UCL Queen Square Institute of Neurology, Faculty of Brain Sciences, University College London London UK; ^2^ Comprehensive Clinical Trials Unit University College London London UK; ^3^ Department of Medical Statistics London School of Hygiene and Tropical Medicine, University College London London UK; ^4^ National Institute for Health Research, University College London Hospitals Biomedical Research Centre London UK

**Keywords:** clinical trials, cognition, frontal assessment battery, secondary progressive multiple sclerosis

## Abstract

**Introduction:**

Cognitive impairment is common in secondary progressive multiple sclerosis (SPMS), with executive dysfunction disproportionately so. The frontal assessment battery (FAB) is a bedside test assessing executive function. This study explores the distribution of FAB scores in a large SPMS cohort and their associations with disability.

**Methods:**

Data were analysed from 294 participants in a cognitive substudy of the MS‐STAT2 trial (NCT03387670). Associations between baseline FAB scores, ambulation status (Expanded Disability Status Scale [EDSS] < 6.0 vs. ≥ 6.0) and other disability measures were assessed using generalised linear models, adjusting for age, education, gender and disease duration. FAB performance was also compared against other cognitive tests (SDMT, CVLT‐II, BVMT‐R).

**Results:**

23.8% of participants scored the FAB maximum of 18; 29.9% scored below the clinical threshold of 16. FAB scores showed moderate correlations with SDMT (*ρ* = 0.46), CVLT‐II (*ρ* = 0.36) and BVMT‐R (*ρ* = 0.43), and participants scoring < 16 were significantly more likely to be impaired across these cognitive domains (*p* < 0.001). Lower baseline FAB scores were significantly associated with higher EDSS, slower T25FW and reduced manual dexterity (9HPT) (all *p* < 0.005) at baseline and longitudinally, with performance comparable to other validated cognitive tests.

**Conclusions:**

We present a large cohort of FAB scores in the SPMS population. Lower FAB scores are associated with both concurrent and future disability and may offer a scalable tool for identifying individuals at greater risk of progression and a robust trial outcome measure.

## 1 Introduction

Cognitive impairment is common in secondary progressive multiple sclerosis (SPMS), with estimated prevalence between 40% and 80%. Within this, executive function—procedural memory of learnt motor and cognitive routines involved in planning, decision‐making, response to feedback, inhibition and flexibility—is disproportionately affected compared to other multiple sclerosis (MS) subtypes [1, 2, 3, 4].

Assessing cognitive deficits in SPMS requires tools that are both sensitive to executive dysfunction and practical for clinical and research applications. The frontal assessment battery (FAB) is a 10‐min bedside test that probes six aspects of executive function (conceptualisation, verbal fluency, motor series, conflicting instructions, inhibitory control and automation). Scores range from 0 to 18 [5]. It has become widely used not just in the early diagnosis and differentiation of behavioural variant frontotemporal dementia but also to demonstrate fronto‐striatal dysfunction in numerous other disease processes [6, 7, 8, 9, 10, 11, 12].

The FAB has been validated as sensitive to frontal lobe dysfunction and concords well with more detailed neuropsychological testing and neuro‐imaging studies [5, 6, 13, 14, 15, 16, 17, 18]. It remains unclear exactly what constitutes an ‘impaired’ performance range on the FAB, with no consensus for a cut‐off score and a relative dearth of normative information. In the original publication outlining the FAB, the mean score for the limited control group (*n* = 42) was 17.3 ± 0.8, leading the authors to suggest that an FAB < 16/18 would be ‘abnormal’ [5]. Scores lower than this are not uncommon in various international studies that have since provided somewhat limited normative data, which is to say it would be misguided to suggest any specific FAB score cut‐off could universally indicate ‘impairment’, particularly in disease processes like MS where it is not yet validated [8, 19, 20, 21, 22, 23, 24]. Increasing age is generally associated with a lower FAB score, while having more years of education is associated with a higher FAB score [21].

Few papers have analysed FAB data in MS populations [4, 25, 26]. The most detailed analysis of the FAB in SPMS comes from the MS‐STAT trial of high dose simvastatin (*n* = 140), where it was used as an independently reported cognitive outcome measure [4]. The MS‐STAT trial demonstrated the sensitivity of FAB to frontal lobe dysfunction in SPMS and found that scores correlated with serum neurofilament light chain, a marker of neuroaxonal injury [27]. To build on this, we used data from the cognitive substudy of the larger MS‐STAT2 trial (*n* = 294) to examine the distribution of FAB scores in SPMS and their association with both cross‐sectional and longitudinal measures of disability. We explored correlations between FAB and other cognitive and clinical outcomes and assessed the ability of FAB scores—both continuous and binary—to predict current and future ambulation status.

## 2 Methods

### 2.1 Study Design and Participants

This study used longitudinal data from the MS‐STAT2 trial, a multicentre, phase 3 randomised controlled trial evaluating high‐dose simvastatin versus placebo in SPMS (NCT03387670) [28]. The trial included participants aged 25–65 years with confirmed SPMS, an Expanded Disability Status Scale (EDSS) score between 4.0 and 6.5 and evidence of ongoing disability progression.

The FAB substudy was conducted only at the trial's lead University College London Hospital NHS Trust (UCLH) site. From a total of 315 participants, 294 were recruited into the FAB substudy and included in this analysis.

Demographic data, baseline FAB scores and baseline *and* 36‐month longitudinal clinical assessments were analysed. As well as the EDSS, the Timed 25‐ft walk (T25FW) and the Nine‐hole peg test (9HPT) were included as they represent validated measures of upper and lower limb motor function, respectively, and are commonly used endpoints in progressive MS trials. Baseline cognitive data were also available in the form of the Brief Visuospatial Memory Test‐Revised (BVMTR), California Verbal Learning Test, Second Edition (CVLT‐II) and the Symbol Digit Modalities Test (SDMT).

### 2.2 Ethics

Ethics approval was obtained from the London Westminster Research Ethics Committee (Ref: 17/LO/1509), and the study adhered to the principles outlined in the Declaration of Helsinki [29]. Written informed consent was obtained from all participants prior to enrolment.

### 2.3 Statistical Analysis

Statistical analyses were performed using R. Descriptive statistics were used to summarise demographic, clinical and cognitive characteristics. FAB scores, which are not normally distributed in healthy controls and lack appropriate population norms for z‐transformation, were treated as raw scores and also dichotomised using a score of 16 as a pragmatic cut‐point to examine associations with disability outcomes, based on existing literature and our cohort distribution.

Cognitive scores from SDMT, CVLT‐II and BVMT‐R were transformed into *z*‐scores using normative data, and cognitive impairment in each domain was defined as a *z*‐score of ≤ −1.5 [30]. The 9HPT was expressed as the reciprocal of the average completion time (1/s), and the T25FW was converted to feet per second to facilitate interpretation.

### 2.4 Correlation Analyses

Spearman correlation coefficients were used to examine the association between FAB scores and other cognitive and clinical measures, including EDSS, T25FW, 9HPT, SDMT, CVLT‐II and BVMT‐R. Differences between ‘higher’ and ‘lower’ FAB groups (≥ 16 vs. < 16, respectively) were explored using Wilcoxon rank‐sum tests, *t*‐tests and chi‐squared tests, depending on the variable type.

### 2.5 Baseline Regression Models

Logistic regression was used to model the association between FAB score and EDSS status (≥ 6.0 vs. < 6.0) at baseline, both as a continuous and binary predictor, adjusting for age, sex, years of education and SPMS duration. Linear regression was also used to assess the relationship between continuous FAB scores and performance on the 9HPT and T25FW, using the same covariates. The relative performance of FAB compared to other cognitive variables (SDMT, CVLT‐II, BVMT‐R) was assessed by constructing separate models for each and comparing model fit using Akaike (AIC) and Bayesian (BIC) information criteria.

### 2.6 Longitudinal Models

We constructed additional logistic regression models examining whether baseline FAB scores predicted ambulation status (EDSS ≥ 6.0) at 36 months. These models adjusted for age, sex, years of education, SPMS duration and treatment allocation (simvastatin vs. placebo) and were repeated with both continuous and binary FAB predictors. As with baseline models, AIC and BIC were used to assess relative model fit compared to other cognitive tests.

## 3 Results

### 3.1 Study Population and Baseline Characteristics

Among the 294 participants included in the FAB substudy (Table 1), the median age was 55 years old (IQR 49–60); 72.4% were female. Median disease duration was 23.0 years (IQR 16.7–30.0), with a median progressive phase duration of 6.7 years (IQR 4.3–9.3). Median EDSS was 6.0 (IQR 5.0–6.5). The median FAB score was 16 (IQR 15–17); 23.8% of participants achieved the maximum possible score of 18/18; and 29.9% (*n* = 88) scored < 16. At baseline, higher FAB scores were significantly associated with better performance across several clinical disability measures. Specifically, FAB scores showed a modest positive correlation with walking speed (timed 25‐foot walk; *ρ* = 0.22, *p* < 0.001) and manual dexterity (Nine‐Hole Peg Test; *ρ* = 0.30, *p* < 0.001), and a negative correlation with disability severity as measured by the EDSS (*ρ* = −0.23, *p* < 0.001).

**TABLE 1:** Demographics, disease characteristics and outcomes.

Baseline variable	Total	By baseline FAB score		
		High (> 16)	Low (< 16)	*p*
*N*	294	206 (70.1%)	88 (29.9%)	NA
Female *N* (%)	213 (72.4%)	148 (71.8%)	65 (73.9%)	0.983
MS duration (yrs, Median IQR)	23.09 (16.66–30.00)	23.21 (16.28–30.48)	23.09 (17.36–30.00)	0.701
SPMS duration (yrs, Median IQR)	6.70 (4.27–9.34)	6.67 (4.41–9.29)	6.81 (4.00–10.00)	0.932
Age (Median, IQR)	55.00 (49.00–60.00)	55.00 (50.00–60.00)	56.00 (48.00–61.00)	0.790
Years of education (Median, IQR)	15.00 (12.00–18.00)	16.00 (13.00–18.00)	14.00 (12.00–17.00)	**0.041**
EDSS (Median, IQR)	6.0 (5.0–6.5)	6.0 (4.5–6.0)	6.0 (6.0–6.5)	**< 0.001**
T25FW (Median, IQR ft/s)	2.07 (1.36–3.10)	2.20 (1.52–3.22)	1.76 (1.07–2.57)	**0.004**
9HPT (Mean ± SD, 1/s)	0.033 ± 0.009	0.035 ± 0.009	0.029 ± 0.009	**< 0.001**
SDMT (Mean ± SD, Z)	−1.695 ± 1.527	−1.314 ± 1.468	−2.587 ± 1.273	**< 0.001**
CVLT‐II (Mean ± SD, Z)	−0.774 ± 1.621	−0.470 ± 1.517	−1.484 ± 1.641	**< 0.001**
BVMT‐R (Median, IQR Z)	0.08 (−0.92–1.51)	0.59 (−0.62–1.80)	−0.76 (−1.40–0.08)	**< 0.001**.

*Note:* Bold *p*‐values are statistically significant, using a threshold of *p* < 0.05. This was done to help readers quickly identify the key findings.

When analysed categorically, participants with FAB scores < 16 performed significantly worse across these domains. Compared to those scoring ≥ 16, they walked more slowly (median T25FW 1.76 vs. 2.20 ft/s, *p* = 0.004), had lower manual dexterity (9HPT: 0.029 vs. 0.035 1/s, *p* < 0.001) and had higher median EDSS scores (6.0 vs. 6.0, *p* < 0.001), despite similar age, gender and disease duration.

FAB demonstrated moderate positive correlations with all other cognitive tests, including the SDMT (*ρ* = 0.46, *p* < 0.001), CVLT‐II (*ρ* = 0.36, *p* < 0.001) and BVMT‐R (*ρ* = 0.43, *p* < 0.001). When using a z‐score cut‐off of ≤ −1.5 to define cognitive impairment in these other cognitive scores, participants scoring < 16 on the FAB were significantly more likely to be impaired on the SDMT (*χ* ^2^ = 33.67, *p* < 0.001), CVLT‐II (*χ* ^2^ = 25.33, *p* < 0.001) and BVMT‐R (*χ* ^2^ = 15.84, *p* < 0.001).

### 3.2 Baseline Predictive Models: FAB and Ambulation Status

Building on the descriptive comparisons, we next assessed whether FAB scores could independently predict ambulation status at baseline, using both continuous and binary models. In a multivariable logistic regression model adjusted for age, sex, years of education and SPMS duration, baseline FAB score (continuous) was a significant independent predictor of ambulation aid use.

**FIGURE 1:**
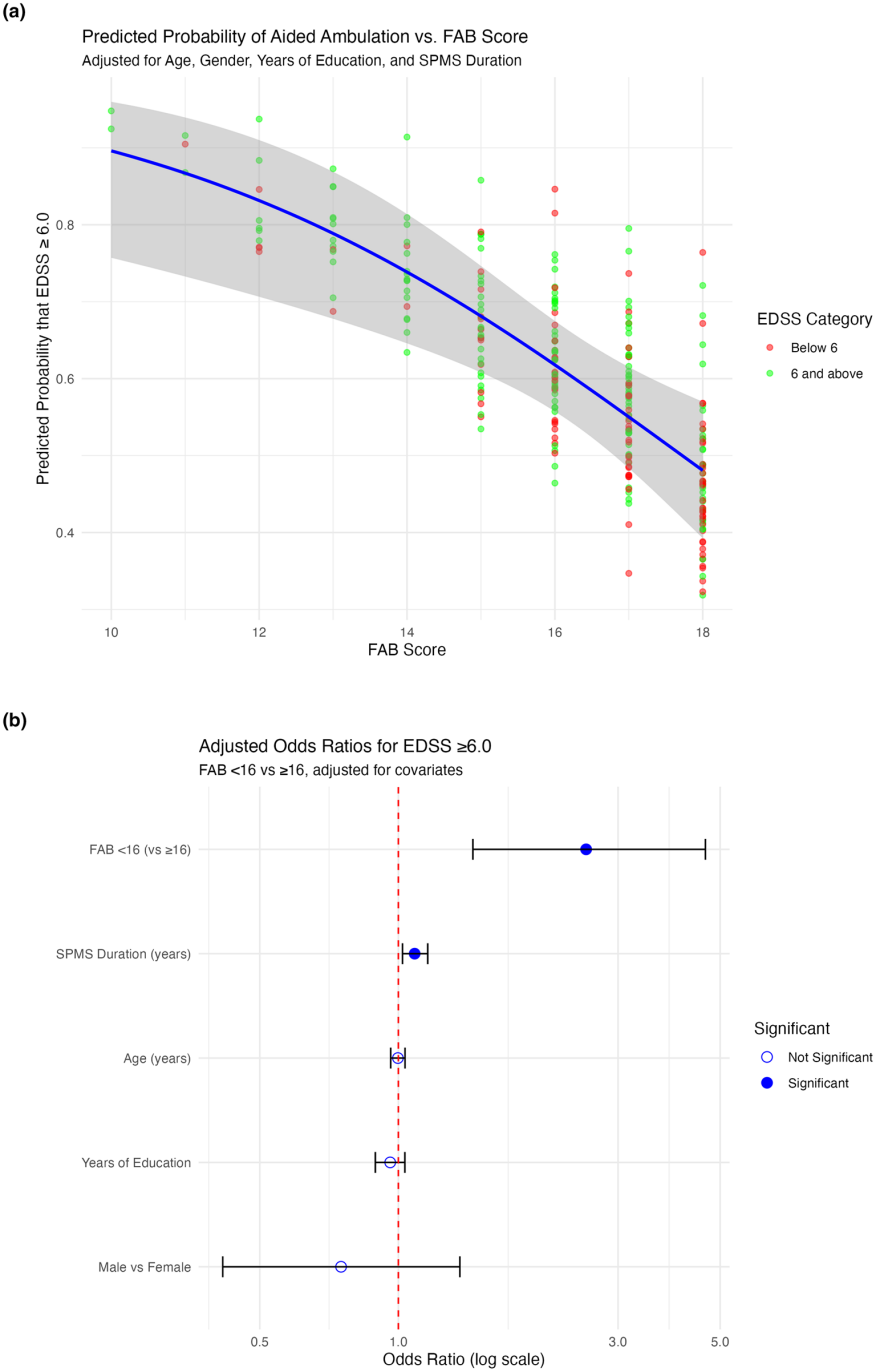
(a) Predicted probability of baseline EDSS ≥ 6.0 versus baseline FAB score; adjusted for age, years of education and SPMS duration. Each point represents an individual, coloured by EDSS category. The blue line represents the logistic regression model's marginal predicted probabilities, with varying FAB score while holding other predictors constant, with the shaded area indicating the 95% confidence interval. (b) Forest plot of odds ratios for requiring ambulation at baseline aid by baseline FAB group. Each point represents the OR, with horizontal lines indicating the 95% confidence intervals (CIs).

Each one‐point increase in FAB score was associated with a 23% reduction in the odds of requiring an ambulation aid (OR = 0.77, 95% CI: 0.65–0.90, *p* = 0.0014; AIC = 367.2; Figure 1a). When dichotomised using our established threshold (< 16 vs. ≥ 16), lower FAB scores were again associated with higher odds of requiring an aid (OR = 2.56, 95% CI: 1.42–4.61, *p* = 0.0015; AIC = 367.7; Figure 1b). These relationships remained robust after adjusting for demographic and clinical covariates. FAB scores demonstrated similar predictive utility to other cognitive tests. The SDMT and CVLT‐II models had marginally better fit (AIC = 361.1 and 361.2, respectively), while the BVMT‐R model showed a higher AIC (374.4). When binary cognitive impairment (*Z* ≤ −1.5) was used, FAB again performed favourably, with a lower AIC (367.7) than CVLT‐II or BVMT‐R.

### 3.3 Longitudinal Associations

Baseline FAB scores remained significantly associated with disability outcomes at 36 months. Higher FAB scores correlated with faster walking speed (T25FW; *ρ* = 0.17, *p* = 0.005), better manual dexterity (9HPT; *ρ* = 0.34, *p* < 0.001) and lower EDSS scores (*ρ* = −0.22, *p* < 0.001). Looking at categorical data (Table 2), compared to those scoring ≥ 16, those scoring > 16 walked more slowly (median T25FW: 1.33 vs. 1.76 ft/s, *p* = 0.032), had reduced manual dexterity (9HPT: 0.03 vs. 0.03 1/s, *p* < 0.001) and exhibited higher disability (median EDSS: 6.5 vs. 6.0, *p* < 0.001).

**TABLE 2:** 36‐month outcomes by baseline FAB score.

Longitudinal (36 month) variable	Total	By baseline FAB score		
		High (> 16)	Low (< 16)	*p*
EDSS (Median, IQR)	6.00 (5.50–6.50)	6.00 (5.50–6.50)	6.50 (6.00–6.50)	**< 0.001**
T25FW (Median, IQR ft/s)	1.66 (0.94–2.79)	1.76 (1.06–2.83)	1.33 (0.59–2.61)	**0.032**
9HPT (Mean ± SD, 1/s)	0.03 ± 0.01	0.03 ± 0.01	0.03 ± 0.01	**< 0.001**
SDMT (Mean ± SD, Z)	44.96 ± 12.89	48.62 ± 10.97	36.35 ± 13.04	**< 0.001**
CVLT‐II (Mean ± SD, Z)	47.82 ± 12.78	50.53 ± 11.13	41.38 ± 14.16	**< 0.001**
BVMT‐R (Median, IQR Z)	20.60 ± 8.33	22.94 ± 7.40	14.93 ± 7.75	**< 0.001**

*Note:* Bold *p*‐values are statistically significant, using a threshold of *p* < 0.05. This was done to help readers quickly identify the key findings.

### 3.4 Longitudinal Models

**FIGURE 2:**
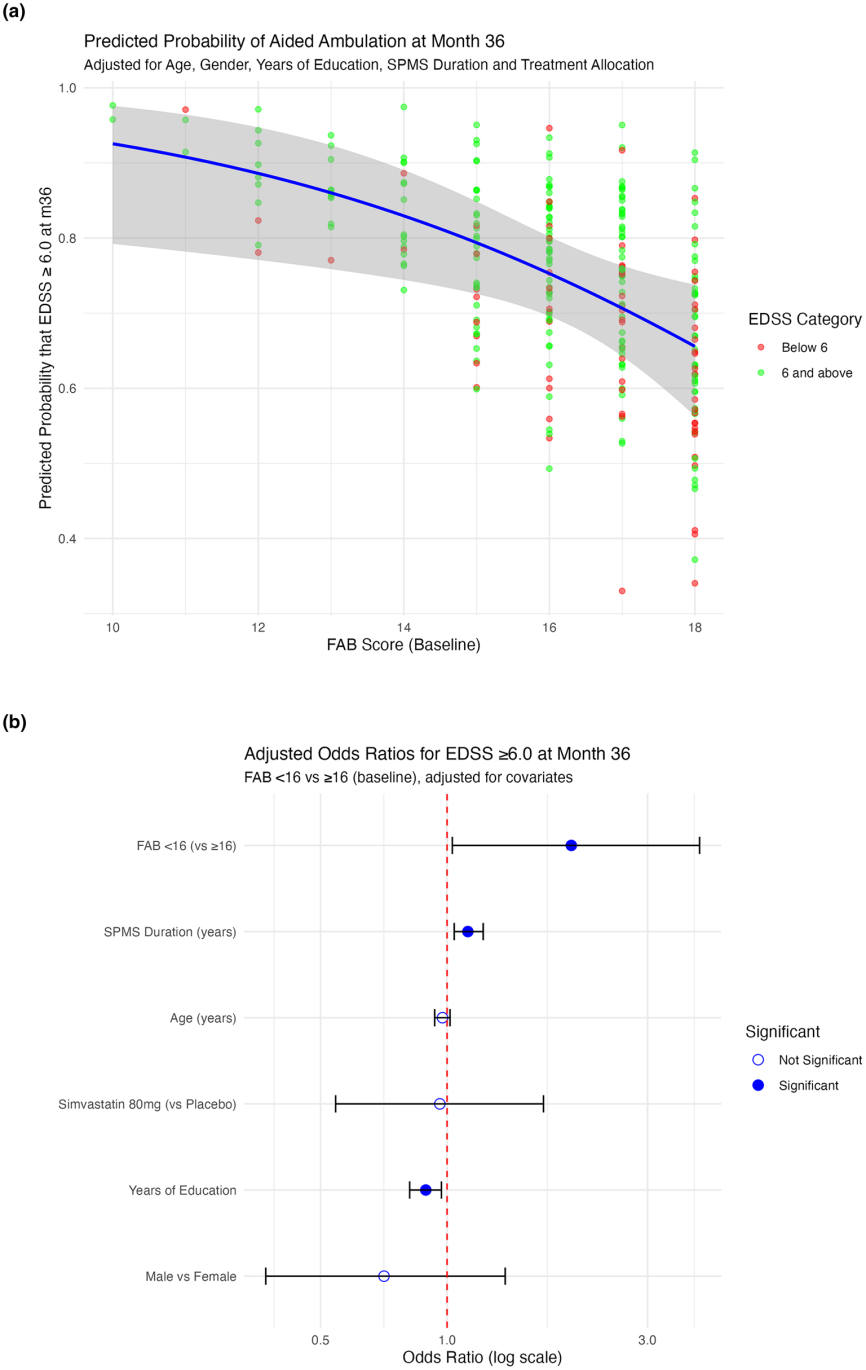
(a) Predicted probability of 36‐month EDSS ≥ 6.0 versus baseline FAB score; adjusted for age, years of education and SPMS duration. Each point represents an individual, coloured by EDSS category. The blue line represents the logistic regression model's marginal predicted probabilities, with varying FAB score while holding other predictors constant, with the shaded area indicating the 95% confidence interval. (b) Forest plot of odds ratios for requiring ambulation aid at 36 months by baseline FAB group). Each point represents the OR, with horizontal lines indicating the 95% confidence intervals (CIs).

To assess the longitudinal predictive value of the FAB, we examined whether baseline FAB scores were associated with ambulation status at 36 months. In an adjusted logistic regression model (Figure 2a), each one‐point increase in baseline FAB score was associated with a 20% reduction in the odds of requiring an ambulation aid (EDSS ≥ 6.0) at 36 months (OR = 0.82, 95% CI: 0.69–0.99, *p* = 0.037; AIC = 303.6). When modelled dichotomously (Figure 2b), participants with FAB scores < 16 had 1.98 times the odds of reaching EDSS ≥ 6.0 at follow‐up (OR = 1.98, 95% CI: 1.00–3.92, *p* = 0.049; AIC = 303.6), adjusted for age, sex, SPMS duration, education and treatment allocation. When compared with other cognitive domains, baseline FAB score demonstrated comparable longitudinal predictive utility. The SDMT model showed slightly better fit (AIC = 292.4), followed by CVLT‐II (AIC = 299.9), FAB (AIC = 301.6) and BVMT‐R (AIC = 302.9). Binary FAB performance was also comparable looking at other cognitive models—SDMT (AIC =297.3), FAB (AIC = 302.1), BVMT‐R (AIC = 303.7), CVLT‐II (AIC = 305.6).

## 4 Discussion

This study represents the largest data set to date assessing FAB scores in individuals with SPMS. It builds on findings from the earlier MS‐STAT trial by further exploring the predictive value of FAB for disability, using both baseline and 36‐month data [4]. A significant proportion of individuals with SPMS demonstrate frontal lobe dysfunction, which is effectively measured with the FAB. FAB scores showed moderate correlations with other cognitive domains, including processing speed, verbal learning and visuospatial memory, and individuals scoring below the clinical threshold of 16 were more likely to be impaired across these domains.

Lower FAB scores were strongly associated with greater disability, including higher EDSS, slower walking speed (T25FW) and reduced manual dexterity (9HPT). These relationships remained significant after adjusting for demographic and clinical confounders, and baseline FAB predicted ambulation status both cross‐sectionally and at 36 months. Compared to other cognitive screening tools (SDMT, CVLT‐II, BVMT‐R), FAB showed comparable predictive value, despite its brevity and narrower cognitive scope. These robust correlations reinforce the impact of executive dysfunction rather than physical disability alone on disability outcomes in MS—the so‐called ‘frontal gait disorder’ [31].

A key advantage of the FAB is its practicality; it takes approximately 10 min to administer, is well tolerated by patients, requires minimal training and does not rely on specialised equipment. It is particularly well‐suited for both routine practice and clinical trials, where efficient and scalable measures are crucial. Its ability to capture executive dysfunction with minimal resource investment adds significant validity as a tool for monitoring progression or assessing therapeutic impact in SPMS trials.

The strengths of this study include the large, well‐characterised, longitudinal cohort of individuals with SPMS, and the rigorous methodology used. There are limitations to consider. The cohort was restricted to individuals with EDSS scores between 4.0 and 6.5, excluding those with very mild or advanced disease, and participants were drawn from a clinical trial population, which may introduce selection bias towards more stable participants—that is, availability for trial visits. Both baseline and longitudinal analyses are of course correlational and do not establish causality.

Future work should examine FAB performance across a broader range of MS phenotypes, explore its relationship with imaging and fluid biomarkers and evaluate longitudinal change in executive function over time. These efforts will be essential to validating the FAB as a scalable, interpretable and effective outcome measure for trials and clinical management in progressive MS.

## Author Contributions

**Charles Wade:** writing – original draft, formal analysis. **Anisha Doshi:** conceptualization. **Sean Apap Mangion:** project administration, investigation. **Tom Williams:** investigation, project administration. **Alessia Bianchi:** investigation, project administration. **Floriana De Angelis:** investigation, project administration. **Sarah Wright:** investigation, project administration. **Nevin** **John:** investigation, project administration. **Alberto Calvi:** investigation, project administration. **Marie Braisher:** project administration. **James Blackstone:** writing – review and editing, supervision, resources. **Jennifer Nicholas:** methodology, formal analysis, writing – review and editing. **Jeremy Chataway:** supervision, conceptualization, writing – review and editing.

## Ethics Statement

The MS‐STAT2 trial was approved by the NHS national research ethics committee (London Westminster Research Ethics Committee, 09/10/2017, ref.: 17/LO/1509).

## Conflicts of Interest

In the last 3 years: J.C. has received support from the Health Technology Assessment (HTA) Programme (National Institute for Health Research, NIHR), the UK MS Society, the US National MS Society, and the Rosetrees Trust. He is supported in part by the NIHR University College London Hospitals (UCLH) Biomedical Research Centre, London, UK. He has been a local principal investigator for a trial in MS funded by MS Canada, as well as a local principal investigator for commercial trials funded by Ionis and Roche. He has taken part in advisory boards/consultancy for Biogen, Contineum Therapeutics, InnoCare, Lucid, Merck, NervGen, Novartis and Roche. J.N. is a principal investigator on commercial MS trials sponsored by Roche, Novartis and Biogen. He has received speaker honoraria from Merck and travel congress sponsorship from Novartis. C.W., A.D., S.A.M., T.W., A.B., F.D.A., S.W., J.N., A.C., M.B. and J.B. have nothing to declare.

## Data Availability

The data supporting the findings of this study are derived from the MS‐STAT2 trial. Access to the anonymised trial data can be made available upon reasonable request to the trial investigators, subject to institutional approvals and data sharing agreements.
